# Incidental Adrenal Enlargement: An Overview from a Retrospective Study in a Chinese Population

**DOI:** 10.1155/2015/192874

**Published:** 2015-03-08

**Authors:** Le-le Li, Wei-jun Gu, Jing-tao Dou, Guo-qing Yang, Zhao-hui Lv, Yi-ming Mu, Ju-ming Lu

**Affiliations:** Department of Endocrinology, Chinese PLA General Hospital, Beijing 100853, China

## Abstract

*Aim*. To investigate incidental adrenal enlargement clinical characteristics and functional status and analyze functional lesion risk factors. *Materials and Methods*. This retrospective study included 578 patients with adrenal imaging features showing enlargement. Incidental adrenal enlargement cases (78) were considered eligible. Demographics, functional diagnosis, adrenal imaging features, and concomitant diseases were analyzed. *Results*. The number of adrenal enlargements and proportion of incidental adrenal enlargement increased each year. Mean patient age was 50.32 years. Thirty-nine cases had unilateral enlargement on the left side and 3 on the right side; 36 had bilateral enlargement. Routine medical checkup was found to have the greatest chance (43.59%) of revealing clinical onsets leading to discovery. Biochemical and functional evaluation revealed 54 (69.23%) cases of nonfunctional lesions, 12 (15.38%) of subclinical Cushing syndrome, 6 (7.69%) of primary hyperaldosteronism, 1 (1.28%) of metastasis, and 5 (6.41%) of unknown functional status. Nodular adrenal enlargement (OR, 7.306; 95% CI, 1.727–28.667; *P* = 0.006) was a risk factor for functional lesions. Age and lesion location were not significant factors. *Conclusion*. Incidental adrenal enlargement is a frequent radiographic finding and is accompanied by diverse clinical factors that require proper evaluation and management. Nodular adrenal enlargement was a risk factor.

## 1. Introduction

With increasing availability, widened indications, and technical refinements of computed tomography (CT) and magnetic resonance imaging (MRI), the number of incidentally discovered adrenal lesions, such as adrenal incidentalomas and incidental adrenal enlargement, is increasing. In a recent study by Tang et al. [1], among a total of 564 eligible CT studies (patients undergoing CT without prior known malignancy, trauma, or endocrine disease), adrenal hyperplasia was detected in 64 cases, giving a prevalence of 11.3%. This indicated that incidental adrenal enlargement had a significant prevalence and has become a common clinical problem.

It should be emphasized that the term incidental adrenal enlargement represents the way the lesion was detected (incidentally), rather than the etiology or diagnosis. It is a common term for a variety of adrenal disorders, but its cause must be properly assessed so that patients needing treatment, such as those with hormone hypersecretion or malignant disease, can receive appropriate care. However, there is a lack of literature on functional status and its follow-up to provide comprehensive insight to these findings.

Patients with incidental adrenal enlargement were evaluated in a tertiary referral hospital with endocrinological departments in China. This study aimed to determine the primary clinical presentation that most frequently leads to the discovery of adrenal enlargement, to evaluate clinical characteristics and functional status of these patients, and to analyze risk factors for functional lesions.

## 2. Materials and Methods

This retrospective study included 578 patients with adrenal imaging features showing adrenal enlargement who were hospitalized at the Department of Endocrinology in PLA General Hospital (Beijing, China) between January 1993 and July 2013. Data retrieved included patient demographics, final functional diagnosis, adrenal imaging features, and concomitant diseases.

Patients were classified as having incidental adrenal enlargement when abdominal imaging was performed for indications unrelated to adrenal disease. Patients with diseases known to cause adrenal enlargement, such as known endocrine disorders which could affect adrenal size, trauma, and underlying malignancy, were excluded. Among all enrolled patients, 78 presented with incidental adrenal enlargement.

The CT imaging technique used was not standardized due to the various clinical indications. However, to be included in their entirety on a maximum of 5 mm section thickness, the upper limit of normal was set as 10 mm for the body of the gland and 5 mm for each limb as documented by Vincent et al. [2]. The type of enlargement, based on subjective evaluation of the adrenal glands, was recorded as either smooth or nodular. Nodular enlargement was diagnosed if the adrenal gland had an irregular contour, contained nodules, and had normal adrenal tissue interspersed between the nodules. Smooth enlargement was defined as enlargement of the gland with a smooth contour and no measureable or diffuse nodules.

After obtaining patient history and physical examination, all patients underwent biochemical evaluation to assess their functional status. Patients with elevated 24-hour urine-free cortisol level (2 times) or those in whom plasma cortisol levels did not decrease after an overnight low-dose dexamethasone test (1 mg DST; cutoff 50 nmol/L) were diagnosed with subclinical Cushing syndrome. Patients with an aldosterone-rennin ratio (ARR) > 20 underwent any 1 of 3 confirmatory tests (saline infusion, captopril challenge, or postural stimulation) to confirm or exclude definitively primary hyperaldosteronism (PA). A nonfunctional lesion was defined as an adrenal gland with no hormonal excess.

Statistical analysis was performed using SPSS Software (version 17.0). Categorical data such as gender and clinical/radiologic features were compared using *χ* ^2^-test or Fisher's exact test. Group data with normal distribution were compared using the Student's *t*-test. Values are expressed as mean ± SD or as number and percentage. A two-sided *P* < 0.05 was considered to indicate statistical significance. Variables that resulted in a *P* < 0.05 in the univariate analyses were entered into logistic regression analysis to assess the risk factors of functional lesions.

The hospital ethics committee approved this study, and written informed consent was obtained from all patients or their parents.

## 3. Results

**Figure 1:**
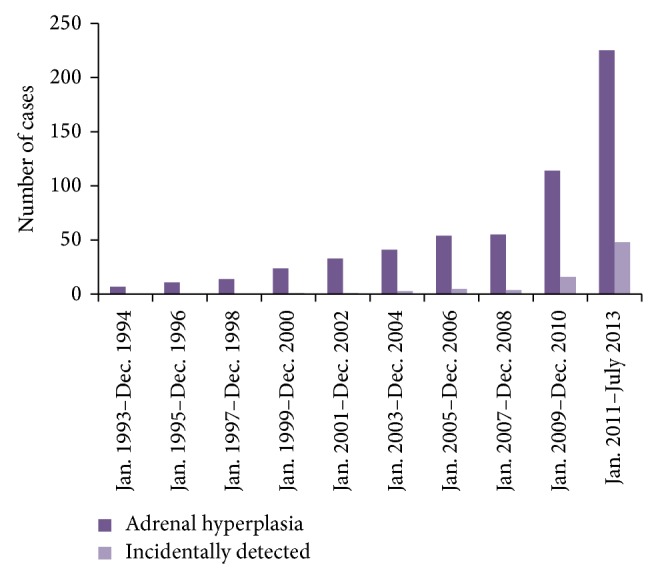
The distribution of cases along with the year.

Of 578 patients with adrenal enlargement, 78 cases (13.49%) were detected incidentally. The distribution of cases by year of discovery is shown in Figure 1. The number of cases gradually increased over time. Every 2 years, the numbers of total cases were 17, 11, 14, 24, 33, 31, 54, 55, 114, and 225, respectively. The numbers of incidental adrenal enlargement cases were 0, 0, 0, 1, 1, 2, 5, 4, 16, and 49, respectively.

In addition, the proportion of incidental adrenal enlargement gradually increased (0, 0, 0, 4.17%, 3.03%, 7.32%, 9.26%, 7.27%, 14.04%, and 21.33%).

Patient clinical characteristics are shown in Table 1. There were 40 men and 38 women. The mean age of the 78 patients was 50.32 years old. 39 cases had unilateral enlargement on the left side and 3 on the right side, and the remaining 36 were bilateral enlargement. As shown in Table 1, routine medical checkup was found to have the greatest chance (43.59%) of revealing clinical onsets leading to the discovery of adrenal enlargement. Predominant complaints included low back pain (10.26%) and abdominal pain (3.85%). In addition, there were 30 (38.46%) cases in which the lesions were incidentally detected during hospitalization for underlying diseases, such as diabetes mellitus, hypertension, and coronary heart disease, among others.

**Figure 2:**
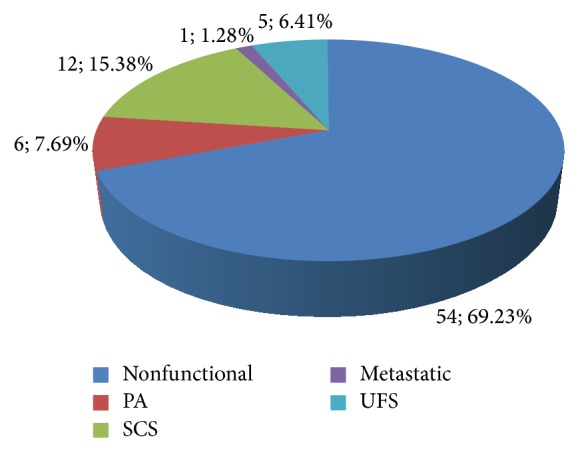
Functional status of patients with incidental adrenal enlargement. SCS: subclinical Cushing syndrome; PA: primary hyperaldosteronism; UFS: unknown functional status.

Biochemical and functional evaluation revealed that 54 (69.23%) cases were nonfunctional and 12 (15.38%) were subclinical Cushing syndrome (SCS); among these patients, 10 cases were diagnosed as AIMAH, the other 2 were diagnosed as adenomas, and they were all confirmed by pathology results. Primary hyperaldosteronism 6 (7.69%), metastatic 1 (1.28%), the primary cancer was gastric cancer. There were 5 patients (6.41%) whose functional status remained unclear because of failure to finish the functional evaluation (Figure 2).

Patients were classified by functional status into 2 groups: functional or nonfunctional. Comparisons of the clinical characteristics of the 2 groups are shown in Table 2. There was no gender difference between the 2 groups. Lesion location was different between the 2 groups (*P* = 0.001). Lesions on the left side were more likely to be nonfunctional. There was also a significant difference (*P* < 0.001) in hyperplasia. Nodular hyperplasia tended to be functional.

Table 3 shows risk factors of a functional lesion. Nodular adrenal enlargement (OR 7.306; 95% CI, 1.727–28.667; *P* = 0.006) was the risk factor for functional lesions. Age and lesion location were not statistically significant factors.

## 4. Discussion

As outlined above, incidental adrenal enlargement is detected with increasing frequency, most likely due to widespread increase in cross-sectional imaging, and is gradually emerging as a common clinical problem. Our study shows that the proportion of incidental adrenal enlargement has gradually increased by year. Mean age at diagnosis was 50.32 years, which is in line with other incidentally detected adrenal findings, namely, adrenal incidentaloma [3]. The increasing age of the general population and a research trend towards more advanced investigations in the elderly population may be contributing to the high detection rate in this age group. Our results indicate that, for the elderly patients, it is essential to place emphasis on these incidental findings.

Tang et al.'s study [1] indicated that, of the total 64 patients, of which 40 (63%) were men and 24 (37%) were women, 43 (67%) cases were bilateral enlargement and 21 (33%) cases were unilateral. In addition, smooth enlargement was more common, in 53 (83%) cases, and together these statistics reflect the likelihood that adrenal enlargement will be bilateral, smooth, and found in men. However, our study did not show this tendency, likely because the research goals and thus, study populations, differed between the 2 studies. Tang et al.'s study aimed to explore prevalence, while the present study aimed to evaluate functional status. In addition, patients enrolled in our study were hospitalized at the Department of Endocrinology. It should be noted that admitting was more or less selective, especially in tertiary referral hospitals, and that economic considerations in parts of China were still a problem.

Clinically, upon discovery of incidental adrenal enlargement, 2 issues arise: functionality and malignancy. In the relevant literature [4–8], adrenal enlargement can result from endocrine disorders, such as adrenocorticotropic hormone- (ACTH-) dependent or independent Cushing syndrome, PA, multiple endocrine neoplasia type 1 (MEN-1), and congenital adrenal hyperplasia. Other potential causes include nonfunctional lesions, defined as a radiographic adrenal enlargement without clinical or biochemical manifestations, inflammation, neoplastic processes, obesity, or depression. A large percentage of incidental cases are due to nonfunctional lesions. Our study is one of the few that attempted to evaluate functional status. Results show that nonfunctional enlargement accounts for 69.23%, which topped the list. 12 patients were found to have subclinical Cushing syndrome (originated from the adrenal gland) and 6 patients were diagnosed with PA. However, the variety of disease spectrum in the study was only moderate, perhaps due, in part, to the limited number of included cases. It is important to note that even though reported prevalence was up to 11.3%, patient referrals to endocrinologists are relatively rare. This is likely related to the poor radiological awareness of this issue and its potential clinical significance.

Finally, we further analyzed risk factors for functional lesions. The results indicated nodular enlargement is a strong risk factor of functional lesions. The clinical significance of lesion location and patient gender is smaller. In the present study, functional adrenal enlargements included subclinical Cushing syndrome and PA. Other published studies have already reviewed the imaging features of the above two disorders. ACTH-independent macronodular hyperplasia (AIMAH) and primary pigmented nodular adrenal hyperplasia often manifest as adrenal hyperplasia. The clinical features of AIMAH tended to be atypical. Thus, adrenal enlargement in some AIMAH patients is incidentally detected. Adrenal CT* manifestation* was characterized by massively, bilaterally enlarged multinodular adrenal glands. Nodules usually distorted and completely obscured the normal adrenal glands and were characteristically “ginger-like” [9]. As for PA, adrenal glands affected by idiopathic hyperaldosteronism (IHA) may be normal on the CT scan or show nodular changes, and small aldosterone producing adenoma (APAs) may be interpreted incorrectly by the radiologists as “IHA” on the basis of CT findings of bilateral nodularity or normal-appearing adrenal glands [10–13]. Imaging features of both disorders are characterized by nodular adrenal hyperplasia. Thus, it is a simple matter to explain why nodular enlargement could be a predictive factor of functional lesions. Meanwhile, this also suggests that if incidentally detected lesions were nodular enlargements, evaluating its functional status should be a priority. In addition, the present study suggests that lesions on the left side were likely to be nonfunctional. Although smaller, this may have certain implications for clinical practice.

There are several limitations in this study. This was a retrospective review of a single center's experience. The sample size was small, which further decreased the power. A lack of clinical and biochemical follow-up was also a limitation. In addition, there was only one patient with malignant lesion in the present study, and thus we were unable to analyze incidence of malignancy.

## 5. Conclusion

Incidental adrenal enlargement is a frequent radiographic finding and it is accompanied by diverse clinical factors that require proper diagnostic evaluation and management. In functional evaluation, nodular adrenal enlargement was found to be an independent risk factor.

## Figures and Tables

**Table 1:** Clinical characteristics of patients with incidental adrenal enlargement.

Characteristic	Value
Gender, male/female	40/38
Age, year	50.32 ± 12.63
BMI, kg/m^2^	26.08 ± 3.69
Location	
Left	39 (50%)
Right	3 (3.85%)
Both	36 (46.15%)
Type of enlargement	
Smooth	39 (50%)
Nodular	39 (50%)
Concomitant disease	
Diabetes mellitus	25 (32.05%)
Hypertension	63 (80.77%)
Reasons for abdominal imaging	
Routine medical checkup	34 (43.59%)
Low back pain	8 (10.26%)
Abdominal pain	3 (3.85%)
Urinary tract disease	3 (3.85%)
Others	30 (38.46%)

BMI: body mass index.

**Table 2:** Comparison of clinical characteristics of functional and nonfunctional lesions.

Characteristic	Functional group	Nonfunctional group	*t*/*χ* ^2^	*P* value
Gender, male/female	12/6	24/30	2.667	0.102
Age, year	54.83 ± 6.84	49.28 ± 13.90	−2.236	0.029
BMI, kg/m^2^	28.01 ± 3.61	25.28 ± 3.67	−2.714	0.009
Location			10.700	0.001
Left	3	34		
Right	2	1		
Both	13	19		
Type of enlargement			12.557	<0.001
Smooth	3	35		
Nodular	15	19		
Concomitant disease				
Diabetes mellitus	8	15	1.725	0.189
Hypertension	15	44	0.000	1.000

**Table 3:** Multiple regression analysis of the risk factors of functional lesions.

Factor	Coefficient	OR	95% CI	*P* value
Age, year	0.020	1.020	0.959–1.085	0.528
Location	0.425	1.530	0.761–3.076	0.233
Type of enlargement				
Nodular to smooth	1.951	7.036	1.727–28.667	0.006

OR: odds ratio; CI: confidence interval.
